# Trajectories of physical functioning and its implication for all-cause mortality in Chinese older people: a large-scale national longitudinal study

**DOI:** 10.7189/jogh.15.04184

**Published:** 2025-06-27

**Authors:** Shisi Shen, Jialu Yang, Ning Ma, Yang Xiong, Tingting Wu, Feng Qin

**Affiliations:** 1 Department of Obstetrics and Gynaecology, Peking Union Medical College Hospital, Chinese Academy of Medical Sciences and Peking Union Medical College, Beijing, China; 2 Division of Prevention and Community Health, National Centre for Cardiovascular Disease, National Clinical Research Centre of Cardiovascular Disease, State Key Laboratory of Cardiovascular Disease, Fuwai Hospital, Peking Union Medical College and Chinese Academy of Medical Sciences, Beijing, China; 3 School of Public Health, Capital Medical University, Beijing, China; 4 The West China Hospital, Sichuan University, Chengdu, China; 5 Chongqing College of Traditional Chinese Medicine, Chongqing, China; 6 Andrology Laboratory, West China Hospital, Sichuan University, Chengdu, China; 7 Department of Urology, West China Hospital, Sichuan University, Chengdu, China

## Abstract

**Background:**

Based on the previous evidence, physical function has been associated with all-cause mortality. However, these studies have been inconsistent. We aimed to conduct trajectory analysis to identify instrumental activities of daily living (IADL) types and estimate their effects on all-cause mortality among older people.

**Methods:**

In the Chinese Longitudinal Healthy Longevity Survey, a total of 13 385 older people aged ≥60 years were included between 2002–18. We employed a group-based trajectory model to determine the IADL trajectories. We fitted a multivariate Cox regression model to evaluate the effects of various IADL trajectories on all-cause mortality. We applied subgroup analyses to explore potential modified effects. We further conducted sensitivity analyses to ascertain the robustness of findings.

**Results:**

Over the 16-year follow-up period, three IADL trajectories were identified, including ‘stable and high function’ (45.7%), ‘rapid increase’ (25.5%), and ‘stable and low function’ (28.7%). The Cox regression model manifested that ‘stable and low function’ was positively associated with high risk of all-cause mortality compared with ‘stable and high function’ (hazard ratio (HR) = 1.33; 95% confidence interval (CI) = 1.25–1.41). Subgroup analyses indicated that this association was also modified by age, income, marital status, social activity, and cognitive impairment (*P* < 0.05). Additionally, our findings remained robust after excluding individuals with chronic diseases or mild cognitive impairment at baseline (*P* < 0.05).

**Conclusions:**

Premature mortality among older people is associated with stable and low IADL function. Additionally, our findings suggested that public health policies should further focus on maintaining functional ability in relatively healthy older people.

An ageing trend has been accelerating in China over recent decades, along with increased mortality and morbidity among older people. It is projected that the proportion of individuals aged >60 years will reach 28% by 2040, driven by rising life expectancy and declining fertility rates [1]. This trend underscores the urgent need for long-term health care for the ageing population.

Physical function includes two primary dimensions – basic activities of daily living (ADL) and instrumental activities of daily living (IADL) [2]. ADL involves fundamental self-care tasks, such as eating, dressing, and mobility, with impairments indicating significant physical decline or advanced disease [3]. In contrast, IADL encompasses complex, cognitively demanding tasks (*e.g.* shopping, financial management) necessary for independent living [4]. The decreasing social ability always indicates a decline in basic care ability [5,6]. Compared to ADL, IADL involves higher-order cognitive and executive functions, making it a more sensitive marker of early functional decline and a more comprehensive assessment of initial functional status. Consequently, IADL was selected as the key independent variable of this study.

Instrumental activities of daily living assessment are crucial for the early detection of individuals at risk of functional decline and mortality. Studies indicate that loss of basic living functions is associated with increased all-cause mortality [7–10]. Notably, meta-analyses revealed that mild cognitive impairment patients show greater IADL impairments than cognitively normal individuals [11]. Given that cognitive decline is a well-established mortality predictor, IADL assessment gains additional clinical significance. This is supported by a 14-year longitudinal study, which further confirmed that dementia significantly elevates mortality risk, with functional decline serving as both a morbidity and mortality predictor [12]. While some studies have examined IADL-mortality associations, findings remain inconsistent due to population heterogeneity [13–15]. Additionally, older adults’ physical function is influenced by sociodemographic factors, health conditions, lifestyle and dietary behaviours [16–20]. It means that individual physical function is determined by some modifiable risk factors from individual preference. Therefore, identifying diverse IADL trajectories is important for next precise intervention.

Prior studies have investigated physical function trajectories (*e.g.* ADL/IADL) in older adults [20–22]. Zhao et al. found four trajectories in Chinese older adults – slow decline, poor function, moderate decline, rapid decline, and stable function [20]. Yang et al. classified IADL trajectories into low-risk, increasing-risk, and high-risk groups [21]. Although these findings highlight the heterogeneity of functional decline, few studies have examined between-group differences in IADL impairment and mortality. Using large-scale longitudinal data (n = 16 064; 17-year follow-up), we assessed the time-varying association between IADL trajectories and all-cause mortality in Chinese older adults.

## METHODS

### Study population

We obtained our study population from the Chinese Longitudinal Healthy Longevity Survey (CLHLS) database, a nationally representative longitudinal study that assesses the physical, mental, and cognitive status, social relationships, lifestyle, and surrounding living environment of older people in China [23]. The CLHLS data sets have been widely applied in geriatric epidemiology research due to their nationally representative sample. The longitudinal survey has been conducted in 1998, 2000, 2002, 2005, 2008, 2011, 2014, and 2018, covering 85% of the elderly population across 22 provinces in China [24]. Initially, it included octogenarians (1998–2000), and then expanded to those aged ≥65 years from 2002 onward. From an initial sample of 16 064 participants, we excluded those aged <65 years (n = 44), those with missing IADL data (n = 53), and those without cognitive assessments (n = 2582) due to cognition's established mortality association [25,26]. Finally, 13 385 older people were included in subsequent analysis between 2002 and 2018 (Figure S1 in the **Online Supplementary Document**).

### Instrumental activities of daily living measurement

Instrumental activities of daily living represent social engagement in daily life for older adults and comprise eight items in the CLHLS questionnaire. These items include visiting their neighbours, cooking a meal, going shopping, washing clothes, walking one km at a time, lifting a weight of five kg, squatting and standing three times without a break, and taking public transportation. Each item has three response options: ‘yes, very limited’, ‘yes, slightly limited,’ and ‘no limited,’ with corresponding scores of three, two, and one, respectively, indicating that higher scores are associated with greater physical limitation. Therefore, the overall scores ranged from eight to 24 as an indicator of physical disability [27].

The IADL scale has demonstrated strong reliability and validity in numerous studies [28–30]. In terms of validity, the scale has shown high construct validity, effectively discriminating between individuals with varying levels of functional ability. Regarding reliability, the IADL scale has consistently exhibited high internal consistency, with Cronbach’s alpha values typically exceeding 0.80 across diverse populations, including older adults [29]. Furthermore, the IADL scale has been validated and widely applied in studies involving elderly populations in China. For instance, in the development and validation of the elderly disability index by Zhang et al., the IADL scale, as a critical sub-component, demonstrated strong discriminative validity in identifying individuals with varying degrees of disability [31]. In addition, IADL was used as a measure of physical functioning in a previous national cohort study exploring physical functioning trajectories and predictors in older adults [32].

### All-cause mortality

During the succeeding surveys in the cohort, the ascertainable date of participants' death was obtained via interviews with family members or local doctors. As previously noted, the cause-specific mortality data in CLHLS may be incomplete and ambiguous, and we did not include them in the current study [33,34]. All interviewees were followed up from baseline until death ascertainment, dropout, and the latest wave of surveys.

### Covariates

We adjusted several potential confounders in the analyses. Demographics included age, sex, residence (rural/urban), education level (illiterate/primary school/high school or above), and marital status (never married or single/married and living with spouse). Lifestyle factors included smoking status (never/former/current), Alcohol drinking status (never/former/current), physical activity (yes/no) and social activity (yes/no). The health status comprised any chronic diseases (hypertension, diabetes, heart disease, stroke, pneumonia, and tuberculosis; yes/no), cognitive function (measured by Mini-mental State Examination (MMSE)), and depression status (assessed with a five-item measure) [35,36] (Appendix 1 in the **Online Supplementary Document**). We developed a directed acyclic graph to visually represent the associations between various covariates and changes in IADL trajectories as well as all-cause mortality (Figure S2 in the **Online Supplementary Document**). Continuous variables were expressed as means (x̄) and standard deviations (SD), and categorical variables as numbers and percentages.

### Statistical analysis

Numerous methodological approaches exist for identifying group-based trajectory changes, including group-based trajectory model (GBTM), latent class growth analysis (LCGA), and mixed-effects models (MEMs). We selected GBTM as the optimal framework for analysing trajectory changes in IADL. LCGA, while useful for univariate trajectory analysis, imposes restrictive assumptions of within-group homogeneity by constraining variability to zero [37]. MEMs are widely employed for longitudinal and multilevel data analysis; they require integration with latent class frameworks (forming growth mixture models) to identify distinct trajectory groups while accounting for both inter-individual and intra-group variations [37]. GBTM is specifically designed to identify heterogeneous developmental patterns in longitudinal data [38,39]. Based on population heterogeneity assumptions, GBTM uses maximum likelihood estimation to classify individuals into trajectory groups and calculate membership probabilities [39]. A pivotal step in GBTM involves determining the optimal number of trajectory groups, balancing model fit and complexity. Typically, the Bayesian information criterion and Akaike information criterion are utilised to assess the model-fitting effect [40,41]. Classification reliability is then confirmed by average posterior probabilities (average posterior probability >0.70) for each trajectory group [21].

The Cox proportional hazards model is a semi-parametric statistical methodology extensively employed in survival analysis to investigate the relationship between a set of covariates and the time-to-event outcomes [42]. We employed Cox proportional hazards models to examine associations between IADL trajectories and all-cause mortality, reporting hazard ratios (HRs) with 95% confidence intervals (CIs). We adjusted Model 1 for age and sex and Model 2 for educational attainment, average income, physical activity, and social activity. We adjusted Model 3 for smoking status, alcohol consumption, physical activity and social activity and Model 4 for depression, MMSE and chronic diseases.

We conducted subgroup analyses to examine the modified effects varied by covariates, including age, sex, years of education, living area, income, smoking status, alcohol drinking, physical activity, social activity, chronic disease status and cognitive impairment. We tested the significant difference of subgroups using the *P*-value from the interaction term.

We also adopted two sensitive analyses to assess the robustness of our findings. First, we excluded people with chronic diseases at baseline from the data sets to estimate the direct effect attributed to IADL impairment. Second, we also excluded people with mild cognitive impairment at baseline to re-analyse the results. We performed all analyses using Stata, version 16.1 (Stata Corporation, College Station, Texas, USA). We considered statistical significance when the two-sided *P*-value was <0.05.

## RESULTS

We included 13 385 older people in the final analysis. The IADL score and age were x̄ = 13.0 (SD = 5.5) and x̄ = 84.9 (SD = 11.5). The score of MMSE was x̄ = 23.0 (SD = 6.9). Included people were more likely to be female (55.9%), live in rural areas (54.3%), never smoke (64.4%), never drink (66.4%), and have no chronic disease (66.4%) (**Table 1**). Three groups had the lowest Akaike information criterion and Bayesian information criterion values, and the average posterior probability of the three subgroups was all >0.7 (**Table 2**).

**Table1:** The characteristics of the included sample*

Variables	Total (n = 13 385)
IADL, x̄ (SD)	13.0 (5.5)
Age in years, x̄ (SD)	84.9 (11.5)
Sex	
*Male*	5906 (44.1)
*Female*	7479 (55.9)
MMSE, x̄ (SD)	23.0 (6.9)
Schooling	
*Illiterate*	8030 (60.0)
*Primary school*	3887 (29.0)
*Higher school*	1468 (11.0)
Living area	
*Rural*	7263 (54.3)
*City*	6122 (45.7)
Marital status	
*Never married*	9050 (67.6)
*Married*	4335 (32.4)
Smoking	
*Never*	8597 (64.4)
*Former*	2157 (16.2)
*Present*	2601 (19.5)
Drinking	
*Never*	8859 (66.4)
*Former*	1617 (12.1)
*Present*	2874 (21.5)
Income	
*Low*	4895 (36.6)
*Median*	4404 (32.9)
*High*	4086 (30.5)
Social activity	
*Never*	11 365 (84.9)
*Sometimes*	1742 (13.0)
*Always*	278 (2.1)
Chronic disease	
*No*	8886 (66.4)
*Yes*	4498 (33.6)
Depression	11.7 (3.3)
Physical activity	
*Never*	7364 (55.1)
*Former*	1332 (10.0)
*Present*	4662 (34.9)

IADL – instrumental activities of daily living, MMSE – Mini-Mental State Examination, SD – standard deviation, x̄ – mean

*Presented as n (%) unless specified otherwise.

**Table 2:** Summary information on the fitness of the IADL trajectory

Group	AIC	BIC	AvePP		
			**Group 1**	**Group 2**	**Group 3**
1	−89 897.41	−89 916.16	1		
2	−85 987.51	−86 017.52	0.94	0.89	
3	−84 307.96	−84 349.22	0.77	0.80	0.88

AIC – Akaike information criterion, AvePP – average posterior probability, BIC – Bayesian information criterion, IADL – instrumental activities of daily living

**Figure 1:**
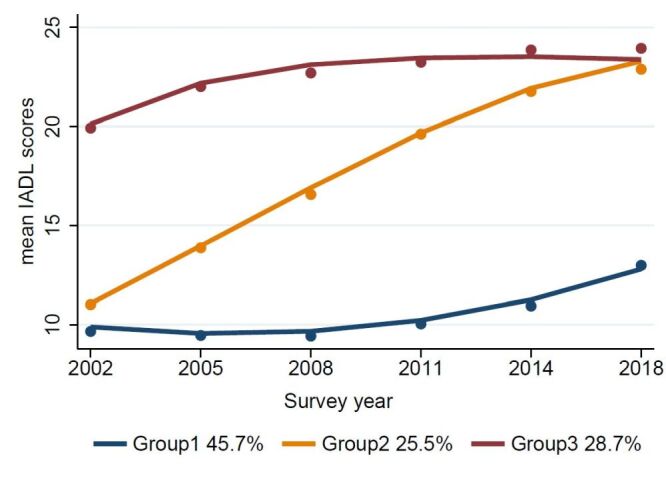
The trajectory plot of IADL subgroup (n = 13 385). Group one indicated ‘stable and high function.’ Group two indicated ‘rapid increase,’ and Group three indicated ‘stable and low function.’ IADL – instrumental activities of daily living.

Three primary IADL trajectories were observed (**Figure 1**). The blue curve showed low IADL scores with a stable trend, and it was named as ‘stable and high function.’ Participants in this group typically exhibit robust self-sufficiency in daily activities, experience minimal physical limitations, and maintain a high level of independence and quality of life, indicating a relatively healthy ageing process. The yellow curve showed a linear growth in IADL scores, and it was named as ‘rapid increase.’ These participants in this group are likely to face increasing activity limitations in the near future, signalling a significant deterioration in physical function. The red curve showed a high IADL scores with stability, and it was named as ‘stable and low function.’ Participants in this group demonstrate substantial and chronic activity limitations, often requiring assistance with most or all instrumental daily tasks, reflecting advanced functional impairment and a reduced capacity for independent living. The ‘stable and high function,’ ‘rapid increase,’ and ‘stable and low function’ accounted for 45.7, 25.5, and 28.7% of IADL, respectively.

**Figure 2:**
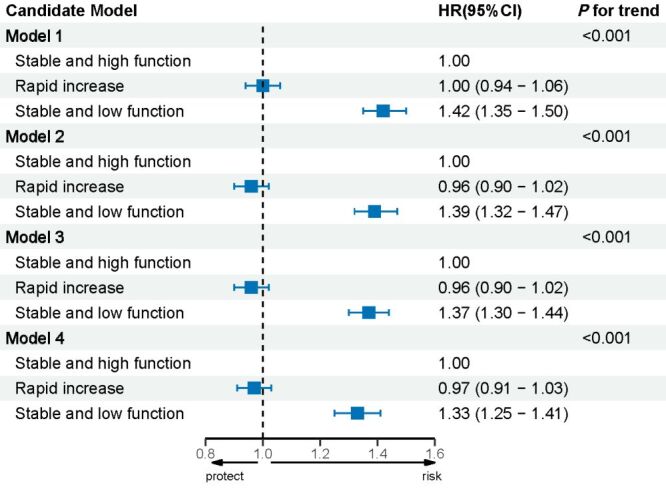
The association between the trajectory group of IADL and all-cause mortality among Chinese older people (n = 13 385). Model 1 adjusted for age and sex. Model 2 further adjusted for education, residence, income and marital status. Model 3 was further adjusted for smoking status, drinking status, social activity, and physical activity. Model 4 was further adjusted for depression, chronic disease, and MMSE grade. CI – confidence interval, HR – hazard ratio, IADL – instrumental activities of daily living, MMSE – Mini-Mental State Examination.

Preliminary analysis in Model 1 reported that ‘stable and low function’ (HR = 1.42; 95% CI = 1.35–1.50) was associated with a high risk of all-cause mortality compared with ‘stable and high function’ (*P* < 0.001). In the fully adjusted Model 4, compared to those with ‘stable and high function,’ individuals with ‘stable and low function’ showed a significant association with higher all-cause mortality (HR = 1.33; 95% CI = 1.25–1.41), but the effects were not identified in ‘rapid increase’ group (*P* > 0.05) (**Figure 2**).

The findings showed that the effects of ‘stable and low function’ were more pronounced in those who were aged <80 years, were married, and urban residents, or those with high income, those with frequent social activity, and those without cognitive impairment (*P* < 0.05) (**Table 3**).

**Table 3:** The association of the trajectory groups of IADL with all-cause mortality stratified by subgroups*

Variables	Trajectory group, HR (95% CI)†		*P*-value	
	**Rapid increase**	**Stable and low function**	**For trend**	**For interaction**
Age in years				<0.001
*≤80*	1.61 (1.44–1.80)	2.38 (1.98–2.86)	<0.001	
*>80*	0.78 (0.72–0.84)	1.16 (1.09–1.24)	<0.001	
Sex				0.463
*Male*	0.98 (0.90–1.08)	1.33 (1.21–1.46)	<0.001	
*Female*	0.95 (0.86–1.04)	1.29 (1.19–1.40)	<0.001	
Years of schooling				0.235
*Illiterate*	0.94 (0.87–1.02)	1.30 (1.21–1.40)	<0.001	
*Primary school*	0.97 (0.86–1.09)	1.37 (1.21–1.54)	<0.001	
*Higher school*	1.17 (0.94–1.47)	1.50 (1.19–1.88)	<0.001	
Living area				0.024
*Rural*	0.92 (0.85–1.00)	1.29 (1.19–1.39)	<0.001	
*City*	1.04 (0.93–1.15)	1.38 (1.26–1.52)	<0.001	
Income in CNY				0.010
*Low*	0.95 (0.85–1.05)	1.31 (1.19–1.44)	<0.001	
*Medium*	0.93 (0.84–1.04)	1.28 (1.15–1.42)	<0.001	
*High*	1.06 (0.93–1.22)	1.42 (1.26–1.60)	<0.001	
Marital status				<0.001
*Never married*	0.86 (0.79–0.93)	1.22 (1.14–1.31)	<0.001	
*Married*	1.24 (1.11–1.39)	1.73 (1.50–2.00)	<0.001	
Smoking status				0.178
*Never smoking*	0.96 (0.88–1.04)	1.33 (1.24–1.44)	<0.001	
*Former smoking*	0.98 (0.83–1.14)	1.36 (1.18–1.57)	<0.001	
*Present smoking*	0.99 (0.86–1.14)	1.19 (1.02–1.39)	0.066	
Drinking				0.890
*Never drinking*	0.99 (0.91–1.07)	1.33 (1.23–1.43)	<0.001	
*Former drinking*	0.95 (0.79–1.14)	1.36 (1.16–1.59)	<0.001	
*Present drinking*	0.90 (0.79–1.03)	1.28 (1.11–1.46)	0.006	
Physical activity				0.684
*Never*	0.98 (0.89–1.07)	1.30 (1.20–1.41)	<0.001	
*Former*	0.87 (0.70–1.09)	1.30 (1.10–1.54)	0.001	
*Present*	0.96 (0.87–1.07)	1.35 (1.21–1.51)	<0.001	
Social activity				0.005
*Never*	0.92 (0.86–0.99)	1.29 (1.21–1.37)	<0.001	
*Sometimes*	1.22 (1.02–1.45)	1.61 (1.29–2.02)	<0.001	
*Always*	2.02 (1.22–3.35)	2.68 (1.21–5.93)	0.002	
Chronic disease				0.644
*No*	0.96 (0.88–1.04)	1.33 (1.23–1.43)	<0.001	
*Yes*	0.98 (0.87–1.09)	1.32 (1.19–1.46)	<0.001	
Cognitive impairment				<0.001
*No*	0.99 (0.92–1.06)	1.39 (1.30–1.49)	<0.001	
*Yes*	0.75 (0.60–0.94)	1.04 (0.90–1.21)	0.302	

CI – confidence interval, HR – hazard ratio, IADL – instrumental activities of daily living

*All models were adjusted for age, sex, education, income, marital status, residence, smoking, alcohol consumption, physical activity, social activity, depression, Mini-Mental State Examination, and chronic diseases (except when these variables served as subgroup variables).

†Stable and high-function group was used as the reference group.

Generally, the findings were consistent with our primary analysis. In the people without chronic disease, we found that ‘stable and low function’ was associated with high all-cause mortality (HR = 1.33; 95% CI = 1.23–1.43), and this finding was established when we excluded people with mild cognitive impairment in another replication (HR = 1.39; 95% CI = 1.30–1.49) (Table S1–2 in the **Online Supplementary Document**).

## DISCUSSION

We designed a seven-wave longitudinal survey to identify the distinct IADL trajectories in the nationally representative ageing population. Three trajectories were ascertained, including ‘stable and high function,’ ‘rapid increase,’ and ‘stable and low function,’ with the proportion of 45.7, 25.5, and 28.7%. Compared with the ‘stable and high function’ group, participants in the ‘stable and low function’ group were significantly associated with high all-cause mortality risk. However, this association does not suggest causality and may be influenced by factors such as age, income, marital status, social activity, and cognitive impairment.

The classification of physical activity varies significantly across studies due to the heterogeneity of the population. In the Chinese longitudinal survey, Zhao et al. reported that the trajectories of IADL could be divided into ‘stable’ (35.4%), ‘slow decline’ (33.0%), ‘poor function with moderate decline’ (8.1%), and ‘rapid decline’ (23.5%) [20]; Yang et al. indicated that three types were sufficient to explain the variation of IADL in ageing population, including ‘low IADL risk’ (41.4%), ‘increasing IADL risk’ (28.5%), and ‘high IADL risk’ (30.5%). Similarly, the 11-year longitudinal study from Taiwan indicated that two trajectory groups could determine the characteristics of IADL among participants aged ≥50 years who had no self-reported IADL limitations at baseline – the late-onset group (67.7%) and the early-onset group (32.3%) [43]. The longitudinal population-based study in community-dwelling frail Dutch aged 60 years demonstrated that IADL function was decreasing with ageing, with deterioration rapidly after self-reported limitation of handling finances, travelling or walking [22]. Our findings found three dynamic trajectories in IADL scores, two stable profiles and one increasing profile. In terms of two stable profiles, one depicted a common class, namely, a majority of participants in relatively complete physical function during the long-term period. Another study illustrated the opposite class, showing that some participants experienced serious physical function loss at baseline, which persisted during the survey cycles. The increasing profile implied partial impairment of IADL at baseline, with this trend gradually deteriorating. These three trajectories are simple and accurate to depict different IADL types, and are convenient for early screening in community-living surroundings.

Besides the early identification of IADL loss, we examined the association between distinct IADL types and all-cause mortality. Our findings align with the previous evidence, indicating that ‘stable and low function’ was significant with a high risk of all-cause mortality compared with ‘stable and high function.’ In Japan, a one-year longitudinal study including 8902 older people found that the association between ADL and mortality showed an inverse direction [7]. The large-scale longitudinal study from the USA among 9447 ageing people found that ADL limitations with ageing could partially account for all-cause mortality, and the statistical significance was ascertained at five and 10 years [8]. In China, a retrospective cohort study investigating the association between community-acquired pneumonia and short-term mortality found that ADL limitations were a substantial predictor related to in-hospital mortality in elderly patients [9]. Moreover, the prospective study from the National Long Term Care Survey in the USA reported that multiple ADL disabilities, coupled with insufficient health care access, contributed to premature death [10]. In our study, we extended the investigation of the relationship between physical activity and all-cause mortality from ADL to IADL. It advances the windows of physical activity screening, and then medical staff could adopt early nursing intervention to improve the quality of life for older people.

Many factors, such as social demographics, lifestyle, dietary habits, health conditions, and living environment, influence physical activity [20,27]. In our subgroup analysis, we found the association between the ‘stable and low function’ group and all-cause mortality was more pronounced in those who were aged <80 years, married, and lived in the city, or those with high income, always engaged in social activities, and no cognitive impairment. Several potential explanations can be proposed. Younger older adults generally maintain higher levels of physical activity. A significant decline in IADL scores in this population may reflect severe limitations in physical functioning, signalling the presence of more serious underlying health conditions [44]. The stronger association observed among married individuals may be attributed to social support systems, including spousal assistance and psychological reinforcement [45,46]. A decline in IADL within this group may indicate disruptions in these support networks or a deterioration in psychological well-being, both of which could contribute to an increased risk of death [47]. In addition, our findings were supported by a previous study using CLHLS. For example, Lv et al. found that the association between cognitive decline and all-cause mortality was stronger among relatively younger older people and those with normal cognitive function at baseline [48]. These novel findings might be explained by several reasons. First, those with IADL limitations might combine a variety of complications, leading to spending more time and effort on medical knowledge and health care. Therefore, their marginal effects are relatively lower than those with robust physical health [21,49,50]. Second, IADL is widely applied in the early identification of physical activity loss [51–54]. Those ageing people with multiple risk factors might experience severe physical impairments, limiting the predictive validity of IADL assessments. Third, surviving older people with multiple risk factors might be more resilient or robust compared to deceased older people. This might imply that the surviving elderly are possibly stronger or more capable of overcoming challenges and hardships in life [55]. In summary, future public health policy should be concerned about relatively healthy individuals, because those people are confronted with a stronger risk of premature death than those with health limitations to a certain extent.

This association between IADL trajectory and all-cause mortality in this study must be contextualised within Chinese cultural norms, family support systems, and institutional policies. Rooted in Confucian filial piety, Chinese society often relies on family support to mitigate functional decline and delay disability progression among older adults, even in cases of significant physical limitations [56,57]. Socioeconomic disparities further influence outcomes, as higher-income households typically access better health care, while low-income and rural populations face substantial barriers. Although China’s health care system has improved through expanded primary care services [58], it still lags behind Western standards in resource allocation and quality [59]. These cultural, economic, and policy factors may buffer or exacerbate the impact of IADL limitations on mortality. While our findings may partially generalise to other regions with similar ageing patterns, Confucian values, and underdeveloped health care systems (*e.g.* parts of Asia, Africa, and Latin America), extrapolation to contexts with distinct cultural, economic, or policy environments requires caution [60].

There are several potential biological mechanisms that may explain the moderating effect of cognitive status on the association between IADL and all-cause mortality. First, the accumulation of inflammation throughout the body mediates both cognitive decline and muscle atrophy, including C-reactive protein and interleukin-6. The underlying pathophysiology may involve a dual role of chronic inflammation, which not only promotes β-amyloid accumulation, a hallmark of neurodegenerative processes [61], but also induces detrimental alterations in skeletal muscle properties, including reductions in maximal muscle strength, explosive power, and rate of force development [62], ultimately progressing to sarcopenia [63]. Second, cognitive decline may impair the brain's capacity to regulate and coordinate muscular activity, resulting in motor dysfunction. Amyloid precursor protein, a type I transmembrane glycoprotein ubiquitously expressed across various tissues and cell types, is predominantly localised at neuronal synaptic sites [64]. In Alzheimer disease, amyloid precursor protein undergoes aberrant proteolytic processing mediated by β-secretase and γ-secretase, leading to the generation of β-amyloid peptides [64]. These β-amyloid peptides are widely recognised as a central pathogenic factor in Alzheimer disease progression, exerting neurotoxic effects on neurons as well as detrimental impacts on skeletal muscle integrity and function [65,66]. Finally, it is noteworthy that approximately 16–32% of individuals with cognitive impairment exhibit comorbid depressive symptoms [67]. These psychiatric abnormalities may further diminish social engagement and physical activity levels, thereby establishing a vicious cycle that exacerbates the decline in physical function.

This study makes several significant contributions to the field. First, by employing advanced longitudinal trajectory analysis, we elucidated the dynamic patterns of IADL functional decline and their robust association with mortality. This methodological approach not only captures the heterogeneity of functional changes but also identifies critical time windows for targeted early interventions. Second, our analysis leveraged data from CLHLS, a nationally representative cohort, which enhances the external validity and generalisability of our findings. Third, through stratified analyses and interaction tests, we revealed population-specific differences in the relationship between IADL trajectories and mortality, offering novel insights for developing personalised health management strategies. Finally, to ensure the robustness of our findings, we conducted two sensitivity analyses, both of which consistently confirmed the stability and reliability of our model.

Several limitations are listed in this study. First, we used all-cause mortality as the main outcome due to the limitation of data sets; unavailable cause-specific mortality data could limit our generalisation of findings and determine the critical cause of death and physical activity loss. Second, detailed dietary data were not accessible, and the proxy variable of the dietary pattern could not be adopted in the baseline questionnaire. Therefore, the associations among dietary patterns, IADL trajectories, and mortality cannot be explored. Third, owing to limited access in the CLHLS, we cannot derive the residential address across every participants, resulting in the lack of mixture pollutant assessment. Fourth, owing to the absence of variables such as access to health care and genetic predisposition in our data set, we are unable to further adjust for the potential influence of these confounders on all-cause mortality. Finally, recall bias may have been introduced due to the retrospective longitudinal study and self-report data.

## CONCLUSIONS

Leveraging a national longitudinal survey, we first investigated the association between IADL trajectories and all-cause mortality among Chinese older people. Typically, three distinct trajectories were identified, including ‘stable and high function,’ ‘rapid increase,’ and ‘stable and low function.’ Then, ‘stable and low function’ is associated with a higher risk of population mortality. In addition, our findings indicated that further public health policy should focus on relatively healthy older people in order to prevent premature death.

## Additional material


Online Supplementary Document
