# Supplementary material for: Trajectories of physical functioning and its implication for all-cause mortality in Chinese older people: a large-scale national longitudinal study
Source: J Glob Health. 2025 Jun 27;15:04184. doi: 10.7189/jogh.15.04184 (PMC12203630; doi:10.7189/jogh.15.04184)

Supplement to: Shen S, Yang J, Ma N, Xiong Y, Wu T, Qin F. Trajectories of physical functioning and its implication for all-cause mortality in Chinese older people: a large-scale national longitudinal study. J Glob Health. 2025;15:04184.

Table S1. The association between the trajectory of IADL and all-mortality excluded those with chronic disease in baseline.

Table S2. The association between the trajectory of IADL and all-mortality excluded those with mild cognitive decline in baseline.

Figure S1. Flow chart.

Figure S2. The hypothetical directed acyclic graph (DAG) used to select potential covariates [a]

[a] Red-marked confounders significantly influence both IADL trajectory changes and all-cause mortality. Blue-marked confounders primarily affect all-cause mortality, while the green-marked confounder mainly impacts IADL trajectory changes.

Appendix S1. Detailed definitions of covariates in our study.

Age (years) was calculated based on the difference between the follow-up year and Georgian calendar dates. The current residence was dichotomized as “Rural area” and “City area.” Education level was divided based on years of schooling (0 years, 1-6 years, and >6 years), referring to illiterate, primary school and high school or above. Smoking and drinking status was defined as “Never,” “Former,” and “Current.” Social activity was defined as whether one took part in social activity, and the item was the score at 1 “never,” 2 “sometimes,” 3 “always.” We coded it as “no”(i.e., never) and “yes” (sometimes and always). The Chinese version of the Mini-Mental State Examination (MMSE) evaluated the global cognitive function. MMSE has four dimensions of cognitive orientation, calculation, recall, and language capacity, with a total of 24 items scoring from 0 to 30, and the higher scores indicate a higher level of dependence for the respondents (Zeng et al. 2010). The elderly who obtained 24 scores and above were defined as “normal cognitive function,” while those who scored less than 24 were evaluated to be “cognitive impairment” (Lei et al. 2020). Six common geriatirc diseases were considered adjusted variables: hypertension, diabetes, heart disease, stroke, pneumonia, and tuberculosis. Depressive symptoms were measured using 5 items as follows: 1. Do you always look on the bright side of things? 2. Are you as happy as when you were young? 3. Do you often feel fearful or anxious? 4. Do you feel the older you get, the more useless you are? And 5. I can make my own decisions concerning my personal affairs. The 5-point response scale to each item ranged from “never” to “always.” Depressive symptoms were the sum of 5 items.

Reference

Yi Z, Vaupel JW. Functional capacity and self–evaluation of health and life of oldest old in China. J Soc Issues. 2010;58(4):733–48.

Lei X, Bai C. Cognitive function and mental health of elderly people in China: findings from 2018 CLHLS survey. China Popul Dev Stud. 2020;3:343–51

Table S1. The association between the trajectory of IADL and all-mortality excluded those with chronic disease in baseline.

Candidate Model	Trajectory Group, HR (95% CI)			Trend p-value
	Stable and high function	Rapid increase	Stable and low function	
Model 1	1.00	0.98 (0.91-1.06)	1.43 (1.34-1.53)	<0.001
Model 2	1.00	0.95 (0.88-1.02)	1.39 (1.30-1.48)	<0.001
Model 3	1.00	0.94 (0.87-1.02)	1.36 (1.28-1.46)	<0.001
Model 4	1.00	0.96 (0.88-1.04)	1.33 (1.23-1.43)	<0.001

Note. Model 1 adjusted for age, sex. Model 2 further adjusted for educational years, income levels, marital status, and residence. Model 3 further adjusted for smoking status, alcohol consumption, physical activity, and social activity. Model 4 further adjusted for depression and MMSE.

Table S2. The association between the trajectory of IADL and all-mortality excluded those with mild cognitive decline in baseline.

Candidate Model	Trajectory Group, HR (95% CI)			Trend p-value
	Stable and high function	Rapid increase	Stable and low function	
Model 1	1.00	1.03 (0.96-1.10)	1.45 (1.36-1.54)	<0.001
Model 2	1.00	0.99 (0.93-1.06)	1.43 (1.35-1.52)	<0.001
Model 3	1.00	0.98 (0.92-1.05)	1.40 (1.31-1.49)	<0.001
Model 4	1.00	0.99 (0.92-1.06)	1.39 (1.30-1.49)	<0.001

Note. Model 1 adjusted for age, sex. Model 2 further adjusted for educational years, income levels, marital status, and residence. Model 3 further adjusted for smoking status, alcohol consumption, physical activity, and social activity. Model 4 further adjusted for depression, and chronic diseases.

Figure S1. Flow chart.

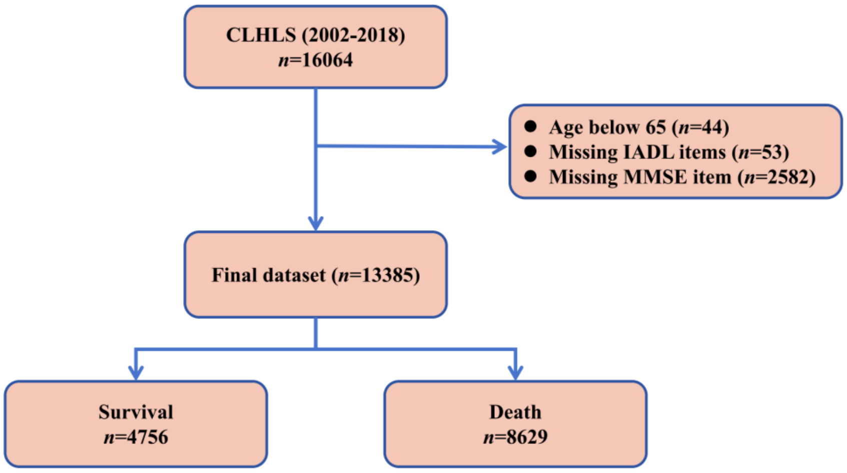

Figure S2. directed acyclic graph (DAG)

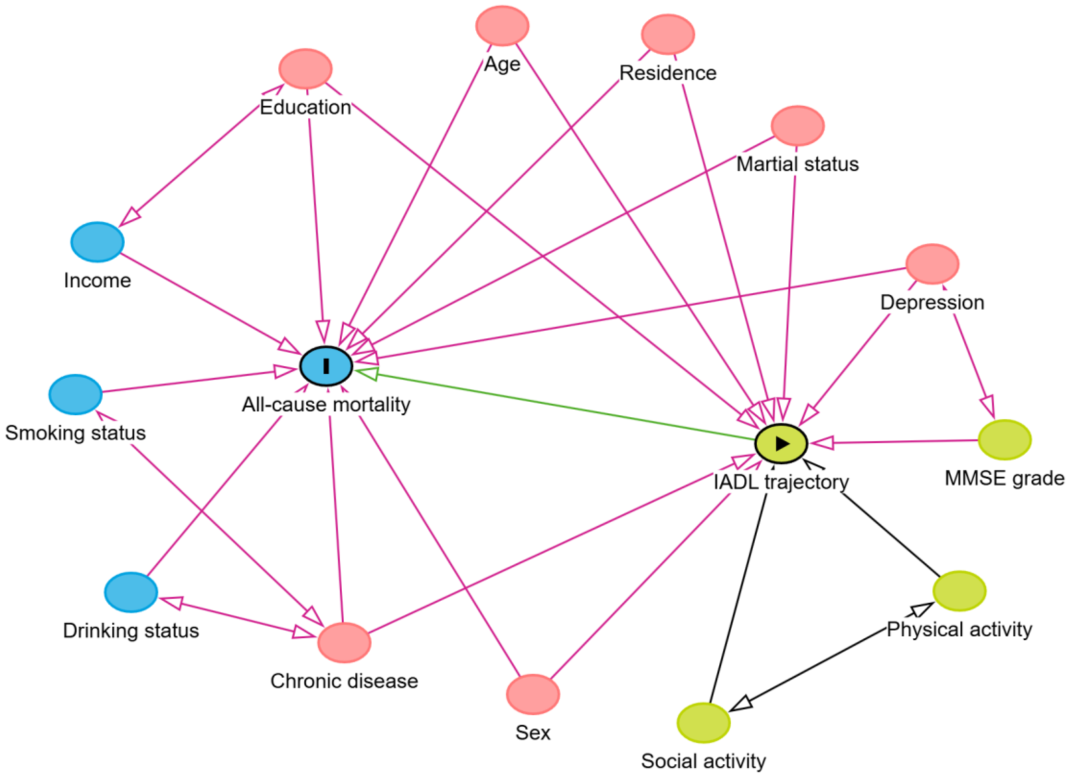

Supplement: Online Supplementary Document [file jogh-15-04184-s001.pdf]
